# Supplementary material for: Volitional Generation of Reproducible, Efficient Temporal Patterns
Source: Brain Sci. 2022 Sep 20;12(10):1269. doi: 10.3390/brainsci12101269 (PMC9599309; doi:10.3390/brainsci12101269)
Supplement: Supplementary file 1 [file brainsci-12-01269-s001.zip › brainsci-1904778-supplementary.pdf]

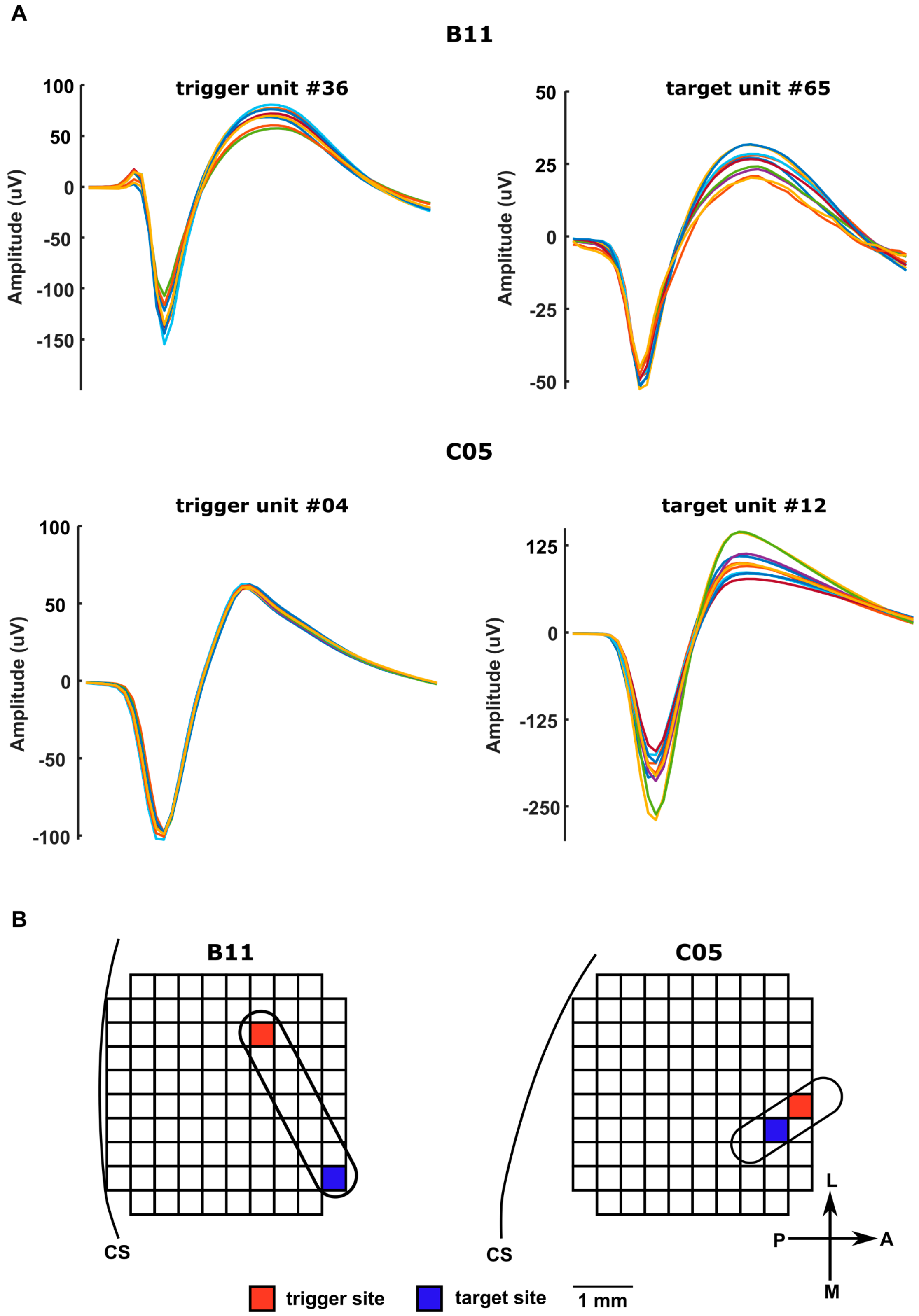

Figure S1. Properties of direct units. (**A**) Consistent waveforms of trigger and target neurons. Different colored curves were the average waveforms of direct neurons in 10 sessions. For C05, the first 5 sessions and the last 5 sessions were taken into consideration. (**B**) Spatial distribution of direct units on the electrodes array.

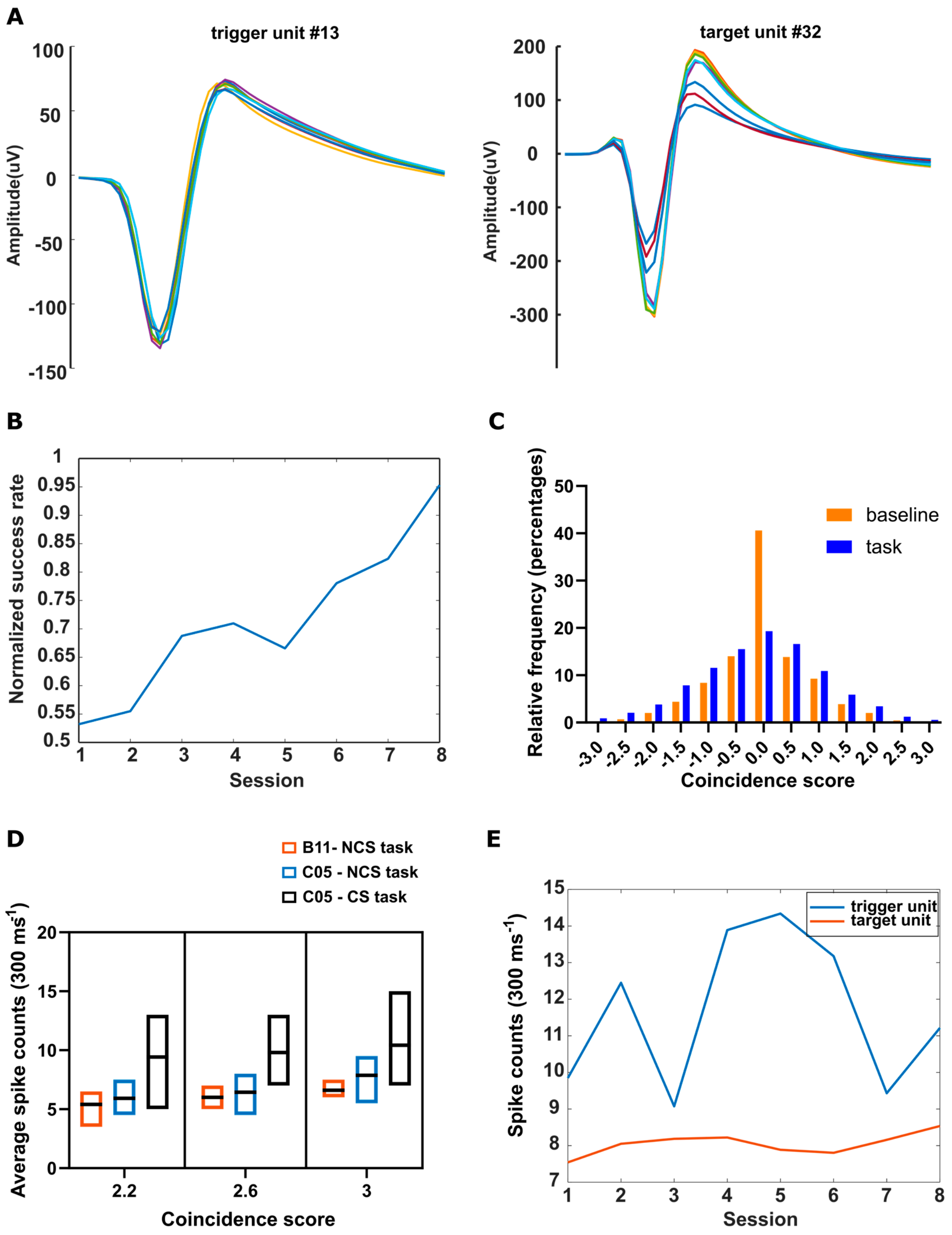

Figure S2. Subject learned to modulate coincidence score in the control experiment. (**A**) Consistent waveforms of trigger unit and target unit. Colored curves were the average waveforms of direct neurons in 8 learning sessions. (**B**) Growing success rate throughout learning. (**C**) The histogram of coincidence score under baseline and task block indicating volitional modulation of coincidence score. (**D**) Spike counts of rewarding neural patterns were lower in the NCS-modulation task than in the CS-modulation (B11: average spike count [NCS task] < average spike count [CS task], $p < 1e-4$; C05: average spike count [NCS task] < average spike count [CS task], $p < 1e-4$). The spike counts were grouped in three blocks according to the coincidence scores of corresponding neural patterns. The black line represented the median. (**E**) The relative stable spike counts of rewarding neural patterns in the CS-modulation experiment justified the result using data from the last session was not a coincidence.

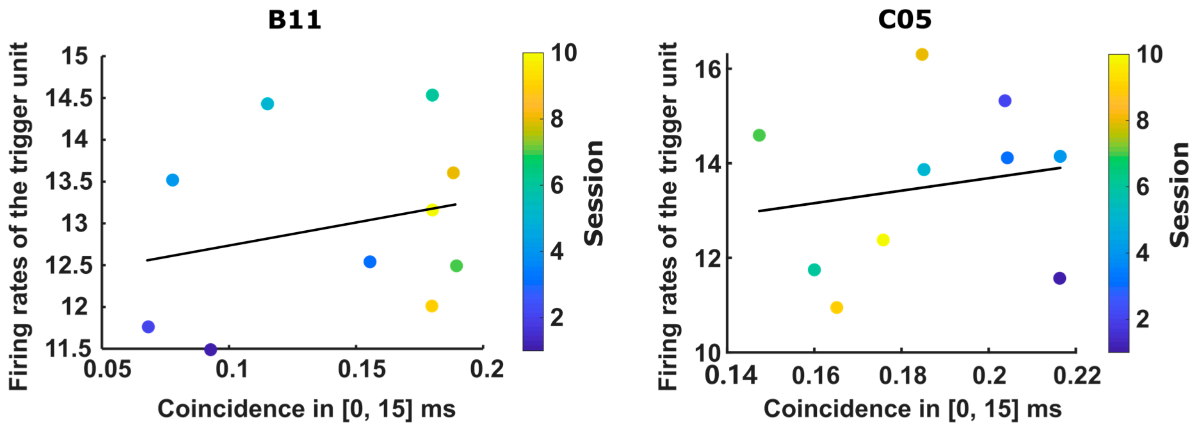

Figure S3. The firing rates of trigger unit was not correlated to the temporal precision across sessions. *Left*: p = 0.48, *right*: p = 0.61.

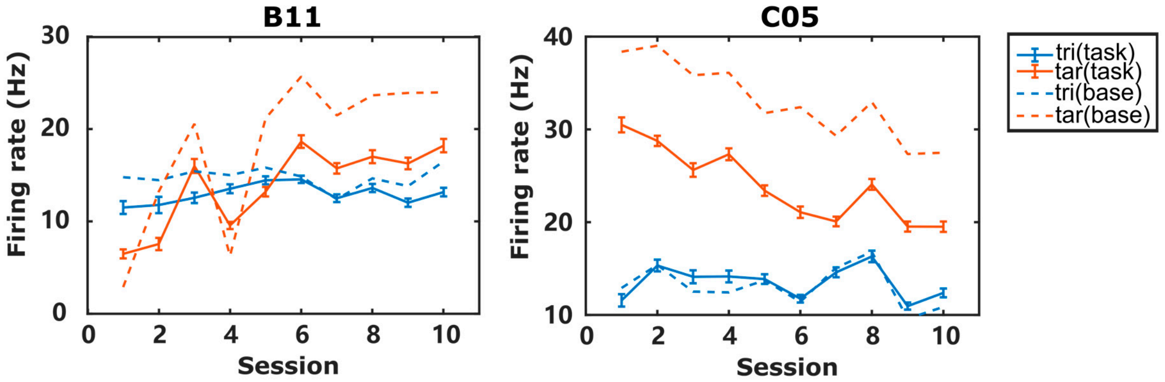

Figure S4. The evolution of firing rates of direct neurons in learning. Color was coded for different neurons, while line type coded for different blocks in one session.

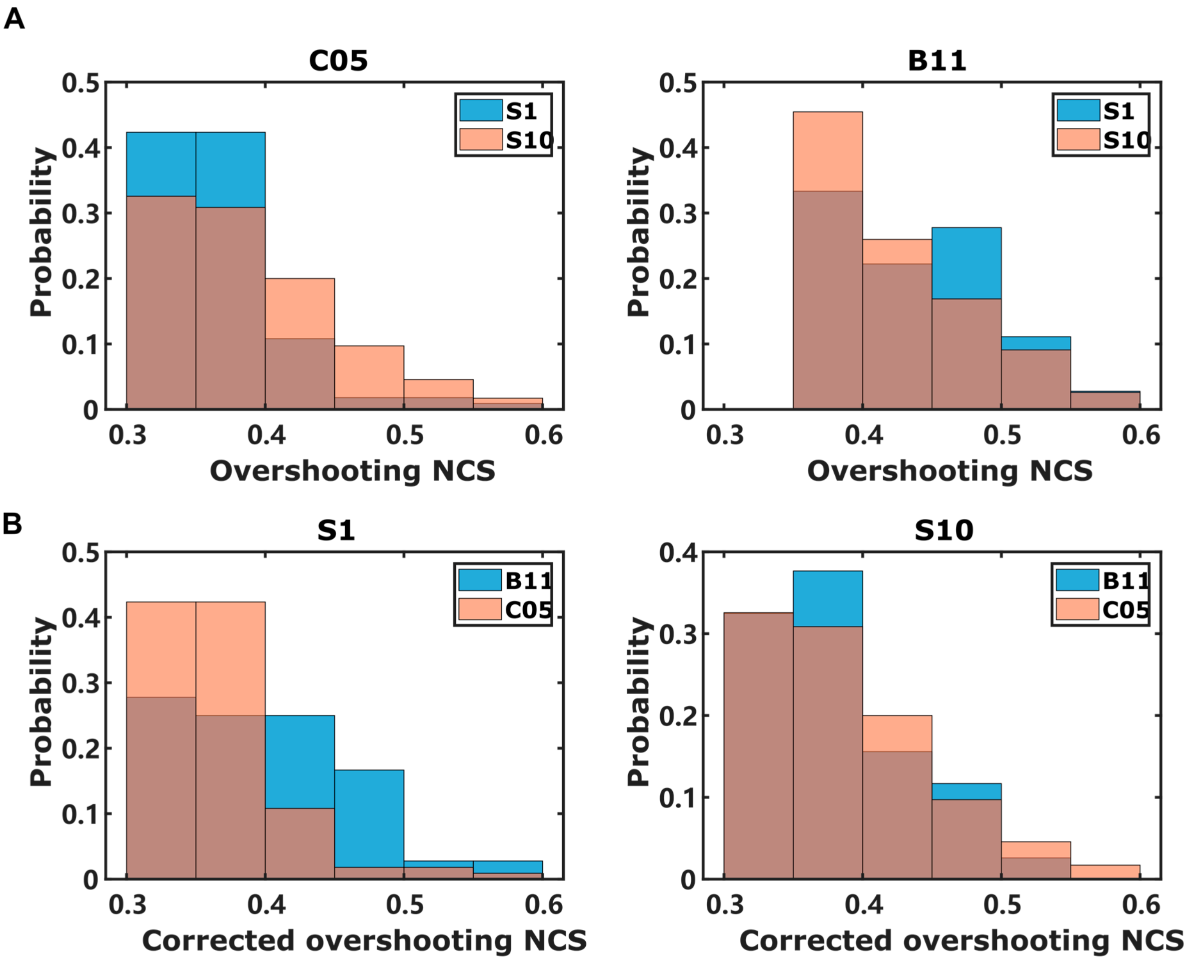

Figure S5. Distribution of overshooting NCS before and after learning. (**A**) Histograms of overshooting NCS from Session 1 (*blue*) and Session 10 (*Orange*). (**B**) *Left*: Distinct histograms of overshooting NCS after correcting in Session 1 (p = 0.003). However in Session 10 (*right*), the histograms were converged (p = 0.65). Subjects are color-coded.
